# Cardiovascular Risk Factors and Antiphospholipid Antibodies in Giant Cell Arteritis-Related Thrombosis

**DOI:** 10.5152/ArchRheumatol.2025.11182

**Published:** 2025-12-01

**Authors:** Georges El Hasbani, Cynthia Crowson, Melissa Snyder, Kenneth Warrington, Matthew Koster

**Affiliations:** Mayo Clinic Faculty of Medicine, Rochester, United States

**Keywords:** Antiphospholipid, giant cell arteritis, thrombosis, visual ischemia

## Abstract

**Background/Aims:**

Giant cell arteritis (GCA), a large-vessel vasculitis, is associated with increased risks of venous and arterial thrombotic events, such as visual ischemia, pulmonary embolism, and ischemic stroke. Traditional cardiovascular risk factors, such as diabetes mellitus, hypertension (HTN), and hyperlipidemia (HLD), might contribute to these events. While antiphospholipid antibodies (aPLs) are sometimes present, their role in GCA-related thrombosis remains uncertain.

**Materials and Methods:**

Seventy-five patients with biopsy-confirmed GCA were recruited, and 1-time blood samples were collected to assess for aPL. Cardiovascular risk factors (e.g., HTN, diabetes, HLD, and atrial fibrillation) at study visit and thrombotic events (pulmonary embolism, deep vein thrombosis, transient ischemic attack, stroke, and visual ischemia) throughout follow-up were abstracted from medical records. Chi-square tests and Cox models were used to evaluate associations between aPLs and these events and their risk factors.

**Results:**

In this cohort of 75 GCA patients, 25 (33%) had at least 1 positive aPL, with anticardiolipin IgM being the most common. Visual ischemia occurred in 19 (25%) patients. There was no significant association between visual ischemia and cardiovascular risk factors, but there was a significant association between anti-beta-2 glycoprotein I IgM and visual ischemia (*P* = .048) as well as between aB2GPI IgG and the development of PE/DVT (*P* = .0035). Overall, 16 experienced a composite thrombotic event, with only aB2GPI IgG associated with higher ischemic event rates.

**Conclusion:**

Although aPL might be prevalent among GCA, there were minimal associations between aPL isotypes and GCA thrombotic events. Further studies are needed to confirm aPL prevalence in GCA and explore anticoagulation’s potential role in patients with visual ischemia and positive aPL.

**Main Points**

- aB2GP1 IgM was more frequent among patients with GCA with visual ischemia than those without visual ischemia
- Phosphatidylserine/prothrombin complex antibodies were not associated with increased risk of arterial or venous thrombosis in GCA
- Prospective studies are needed to evaluate antiphospholipid antibodies at baseline and during follow in GCA patients to determine the overall prevalence of positive findings and the associated impact on vascular features and outcomes

## Introduction

Giant cell arteritis (GCA), a form of large vessel vasculitis, is associated with venous and arterial thrombotic events.^1,2^ Visual ischemia (VI) is a well-known and established complication resulting from the involvement of inflammatory steno-occlusive disease of the ophthalmic and/or posterior ciliary arteries.^3^ The GCA has also been associated with venous events including pulmonary embolism (PE) and deep vein thrombosis (DVT),^4^ while non-ocular arterial events encompass myocardial infarction and ischemic stroke.^5^

Patients with GCA have an increased risk of developing cardiovascular risk factors, such as diabetes mellitus (DM), hypertension (HTN), and hyperlipidemia (HLD), especially after the initiation of treatment.^6^ Antiphospholipid antibodies (aPLs) may be detected in various autoimmune rheumatic and musculoskeletal diseases, potentially contributing to both arterial and venous thrombotic events.^7^ Antiphospholipid antibodies have been detected in patients with GCA with debatable contribution to thrombosis.^8^

This study assessed 75 patients with biopsy-proven GCA for thrombotic events, including VI, PE, DVT, transient ischemic attack (TIA), and ischemic stroke. In addition, the main cardiovascular risk factors in these patients, such as HTN, DM, HLD, and atrial fibrillation (Afib) were examined. Furthermore, the presence and levels of aPL (anticardiolipin (aCL) IgG and IgM, anti-beta-2 glycoprotein I IgG and IgM, and antiphosphatidylserine prothrombin IgG and IgM) were evaluated among these patients. The aim was to assess the prevalence of cardiovascular risk factors and aPL among patients with GCA who experienced a thrombotic event.

## Methods

Patients with biopsy-confirmed GCA, without known additional co-existing rheumatic disease, were recruited in this cross-sectional study from the rheumatology division at Mayo, Rochester, Minnesota, USA from September 2008 through October 2013. This study was reviewed and approved by the Institutional Ethics Committee of Mayo Clinic, under protocol number 08-004669. All procedures performed in this study involving human participants were in accordance with the ethical standards of the institutional and/or national research committee and with the 1964 Helsinki Declaration and its later amendments or comparable ethical standards. Written consent was obtained at the time of sample collection. Plasma samples were collected at the time of recruitment and cryopreserved for future research testing. Standard laboratory parameters were clinically performed at the time of research plasma draw, including complete blood count with differential, erythrocyte sedimentation rate, C-reactive protein, and creatinine. Patients were treated and followed in rheumatology per standard practice but were not assigned to specified follow-up visits or durations. The study complied with the Declaration of Helsinki and was reviewed and approved by the institutional review board (08-004669). All patients signed informed consent prior to recruitment. Seventy-five patients with an established diagnosis of GCA were included in this study and provided a plasma sample for cryopreservation and future testing. Testing for aPL was performed on their enrollment blood draw. The aPLs tested included anticardiolipin (aCL) IgG and IgM, anti-beta-2 glycoprotein I (aB2GPI) IgG and IgM, and antiphosphatidylserine prothrombin (aPS/PT) IgG and IgM. The cut-off value for aPS/PT (IgG and IgM) positivity performed by standardized ELISA was 30 units.^9^ The cutoff for aCL and aB2GPI positivity performed by standardized ELISA was 40 units.^10^ The treatment profile of these patients at the time of inclusion was assessed, and their charts were reviewed to note the presence of HTN, DM, HLD, or Afib documented at the time of sample collection or follow-up clinical visits.

Imaging-proven thrombotic events, including PE, DVT, and stroke, were recorded if they occurred at or after GCA diagnosis. Transient ischemic attack was diagnosed clinically by the assessment of a neurologist. Visual ischemia (double vision, transient or permanent loss of vision), as diagnosed by a comprehensive ophthalmological examination, was noted if it had ever occurred and was attributable to GCA.

Comparisons between groups for categorical variables were performed using Fisher’s Exact test when n < 5 was present or chi-square tests otherwise. Continuous variables were compared between groups using rank-sum tests. Cox proportional hazards models were used to examine associations between variables measured at sample collection and long-term outcomes. Long-term outcomes included composite outcomes for the first of PE or DVT, the first of TIA or ischemic stroke, and first of any of PE, DVT, TIA, or ischemic stroke. Given the limited sample size, these models were univariable except for 1 instance where adjustment for HLD was performed. *P* values less than .05 were considered to be statistically significant. Adjustment for multiple comparisons was not performed because the analyses were considered to be exploratory and the aPL antibodies are correlated, so the tests are not independent. However, the Bonferroni correction would consider *P* values less than .05/6 = .008 for individual aPL antibodies to be statistically significant.^11^ Analyses were performed using SAS version 9.4 (SAS Institute, Cary, NC, USA).

## Results

The study included 75 patients: 60 (80%) women and 15 (20%) men. Mean (±SD) age at inclusion was 75.0 ± 7.3 years. Sixty patients were on prednisone at study enrollment with a median dose of 10 (IQR: 6.5, 55) mg/day. Twenty-five (33%) patients had at least 1 aPL antibody positive; 16 single positive, 7 double positive, and 2 triple positive. The aCL IgM was the most common positive study (n = 14), followed by aCL IgG (n = 6), PS/PT IgM (n = 5), PS/PT IgG (n = 4), aB2GP1 IgM (n = 4, and aB2GP1 IgG (n = 3. The mean disease duration from GCA diagnosis to blood draw was 29.1 months for all patients. The difference in mean disease duration prior to blood draw between patients who were positive or negative for aPL was not statistically significant (39.9 ± 40.8 vs. 23.7 ± 32.5 months, *P* = .098).

Out of the 75 patients, 19 (25%) experienced at least 1 episode of VI (see Table 1) attributable to GCA. The median prednisone dose at study enrollment was similar between patients with (20 mg/day; IQR 10-60) and without (10 mg/day; IQR: 5-40) a history of GCA-associated VI (*P* = .11). There were no statistically significant differences in the prevalence of HTN, DM, HLD, or Afib between those who experienced VI and those who did not. There was no difference between having any aPL study positive among those with VI (8/19; 42%) and those without VI (17/56; 30%; *P* = .35). However, when comparing the presence of individual aPL antibodies aB2GPI IgM was found to be associated with the presence of VI (*P* = .048). There was no statistical relationship between the titer of aPL and the presence of VI.

Nine patients experienced an episode of PE or DVT during a median follow-up of 9.7 (25th-75th percentile: 5.6-14.9) years. There was no statistically significant association between the occurrence of PE/DVT and any of the risk factors: HTN, DM, HLD, or Afib. There was no association between having any aPL study positive and the development of PE/DVT (*P* = .58). However, when comparing the presence of individual aPL antibodies aB2GPI IgG was found to be associated with the development of PE/DVT (Hazard ratio [HR]: 14.46; 95% CI: 2.41-86.85; *P* = .0035). Ischemic stroke and TIA occurred in 9 patients during follow-up. There was no statistically significant association between the occurrence of stroke/TIA and any of the risk factors: HTN, DM, HLD, or Afib. Antiphospholipid antibodies was not associated with an increased risk of developing ischemic stroke or TIA. Overall, 12 patients in this cohort were initiated on anticoagulation, 2 prior to the date of serum sample collection and 10 patients after. Only 1 patient in the cohort met formal classification criteria for antiphospholipid antibody syndrome and was started on warfarin.

Overall, 16 patients in the entire cohort experienced at least 1 arterial or venous thrombotic event during follow-up. There was a statistically significant association between the presence of aB2GPI IgG and the development of any ischemic event (HR: 7.05; 95% CI: 1.42-35.00; *P* = .017), which was slightly attenuated after adjustment for HLD (HR: 5.39; 95% CI: 1.08-26.96; *P* = .040).

## Discussion

The incidence of venous thromboembolic events in a cohort with GCA is estimated at 13.3 per 1000 person-years.^4^ There is an almost 2.5-fold increase in the risk of venous thromboembolic events among patients with GCA.^4^ In this study, 43% of the cohort had at least 1 venous or arterial thrombotic event. Due to the percentage of venous and arterial thrombotic events seen in GCA, it is important to consider etiologic reasons for this occurrence.

Data on the association between traditional thrombotic risk factors and the occurrence of venous thrombosis remain inconclusive. Some evidence suggests that traditional cardiovascular risk factors, particularly essential HTN, increase the risk of severe ischemic events among patients with GCA.^12^ However, other studies indicate that no specific traditional risk factor has been definitively linked to an increased occurrence of venous thromboembolic events in this patient population.^13^

A few earlier reports have demonstrated that different aPL subtypes are prevalent in GCA and may become negative after treatment, although the contribution to thrombotic events was not clear.^14^ In this study, aB2GPI IgM positivity was found to be significantly associated with VI, an association not previously observed in the literature. Despite limited data, some reports suggest that early use of systemic anticoagulation, such as warfarin or intravenous heparin, in combination with glucocorticoids, may improve outcomes—particularly visual impairment—in patients with GCA who have positive aPL.^15^ Visual ischemia related to GCA is a medical emergency, requiring prompt initiation of treatment. It remains undetermined whether the detection of aPL in patients with GCA is transient due to an active inflammatory state or due to chronic immunologic disturbance. Therefore, the importance of checking aPL in patients with VI associated with GCA remains uncertain because it is unknown whether a positive result should prompt a change in treatment approach. In order to determine the persistence of aPL positivity, aPL should be checked 2 times separated by a minimum of 12 weeks within a span of 3 years. In order to understand more fully the presence of aPL and its short- and long-term impacts on GCA, a more systematic approach is needed. First, an estimation of prevalence at the time of diagnosis prior to treatment with repeat testing during follow-up would be required. Second, among patients with aPL detected, well-designed studies evaluating the use of anticoagulation and its impact on preventing or regaining visual function would be necessary, prior to considering use in clinical practice.

Prior studies evaluating arterial and venous thrombosis in GCA have focused on lupus anticoagulant, aCL, and aB2GPI antibodies. Notably, this is the first study to evaluate the prevalence of PS/PT antibodies in patients with GCA. The detection frequency of these antibodies was low, with no associated increased risk, suggesting that the additional utility of checking these antibodies may be limited.

This study must be interpreted in the context of its limitations. First, the sample size was limited. Second, this study was based on stored serum samples collected for a biorepository. Lupus anticoagulant was not assessed at the time of initial sample collection and could not be tested on stored serum, so the presence or absence of lupus anticoagulant in this cohort is unknown. Third, the majority of patients in the cohort were receiving glucocorticoids at the time of study enrollment, which may have influenced aPL positivity or levels of positivity. Fourth, aPL testing was performed on the study entry sample without repeat samples available for testing to confirm persistent antibody positivity. Indeed, 2 consecutive positive aPL results are required, separated by 12 weeks and drawn within 3 years, to formally meet the clinical criterion for APS, which carries known risk of vascular thrombosis. Nevertheless, among patients with chronic rheumatic disease, such as GCA, it is possible that certain aPL antibodies may confer a higher risk than others for arterial compromise, specifically VI. Further testing to evaluate this possible association is needed.

aB2GP1 IgM aPL was more frequent among patients with GCA with VI than those without VI. Traditional cardiovascular risk factors were not associated with increased risk of VI or DVT/PE, and only HLD was observed to be associated with the composite arterial or venous thrombosis group. Further prospective studies with systematic laboratory evaluation at the time of diagnosis are needed to determine the overall prevalence of aPL in patients with GCA. Among patients with positive aPL, repeat assessments are required to understand whether these antibodies persist in a subset or are only transiently observed. Investigation into the role of anticoagulation among patients with GCA with positive aPL and VI is warranted.

## Figures and Tables

**Table 1.** Characteristics of Patients with Giant Cell Arteritis in Relation to Presence or Absence of Visual Ischemia

Characteristic*	No Visual Ischemia (n = 56)	Visual Ischemia (n = 19)	Total (n = 75)	*P*
Age, years, mean (±SD)	74.4 (±6.9)	76.5 (±8.3)	75.0 (±7.3)	.15
Sex, female	46 (82%)	14 (74%)	60 (80%)	.43
Hypertension	42 (75%)	13 (68%)	55 (73%)	.58
Diabetes mellitus	11 (20%)	3 (16%)	14 (19%)	.71
Hyperlipidemia	39 (70%)	15 (79%)	54 (72%)	.44
Atrial fibrillation	20 (36%)	6 (32%)	26 (35%)	.74
aPS/PT IgG	3 (5%)	1 (5%)	4 (5%)	1.00
aPS/PT IgM	4 (7%)	1 (5%)	5 (7%)	1.00
aB2GPI IgG	2 (4%)	1 (5%)	3 (4%)	1.00
aB2GPI IgM	1 (2%)	3 (16%)	4 (5%)	.048**
aCl IgG	4 (7%)	2 (10%)	6 (8%)	.64
aCl IgM	10 (18%)	4 (21%)	14 (19%)	.74
aPL total	17 (30%)	8 (42%)	25 (33%)	.35
Composite aPL				.74
Single	11 (20%)	5 (26%)	16 (21%)	
Double	5 (9%)	2 (10%)	7 (9%)	
Triple	1 (2%)	1 (5%)	2 (3%)	
Prednisone use	47	13	60	
Dose, mg/day, median (IQR)	10 (5, 40)	20 (10, 60)	13.8 (5.5, 40)	.11

aB2GPI, anti-beta-2 glycoprotein I; aCL, anticardiolipin; aPL, anti-phospholipid antibodies; aPS/PT, antiphosphatidylserine prothrombin; IQR, interquartile range; SD, standard deviation.

*Values in table are n (%) unless otherwise specified. *P*-values were obtained using Fisher’s Exact test when there was a value with n < 5, otherwise the chi-square test was used.

**Statistically significant.

## Data Availability

The data that support the findings of this study are available on request from the corresponding author.
